# [3-Bromo-2-(3-fluoro­benz­yloxy)phen­yl]boronic acid

**DOI:** 10.1107/S1600536809033376

**Published:** 2009-08-26

**Authors:** Kinga Kacprzak, Tomasz Klis, Janusz Serwatowski

**Affiliations:** a Physical Chemistry Department, Faculty of Chemistry, Warsaw University of Technology, Noakowskiego 3, 00-664 Warsaw, Poland

## Abstract

In the title compound, C_13_H_11_BBrFO_3_, the dioxy­boron fragment is close to co-planar with the benzene ring to which the B atom is connected [dihedral angle = 8.96 (4)°]. The dihedral angle between the two benzene rings is 14.8 (2)°. One of the OH groups is engaged in an intra­molecular O—H⋯O hydrogen-bonding inter­action. The second OH group is involved in inter­molecular hydrogen bonding, forming a centrosymmetric dimer. The F atom and the corresponding *meta*-H atom are disordered over two positions in a 0.675 (6):0.325 (6) ratio.

## Related literature

For general background to the applications of boronic acids and aryl-benzyl ethers, see: Bien *et al.* (1995 ▶); Dai *et al.* (2009 ▶); Miyaura & Suzuki (1995 ▶). For the structural characterization of a related boronic acid derivative, see: Serwatowski *et al.* (2006 ▶).
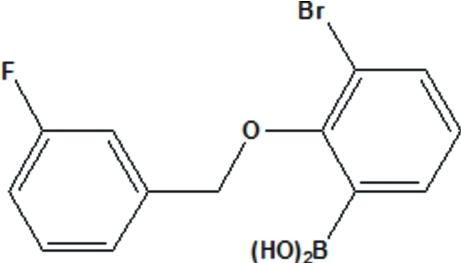


## Experimental

### 

#### Crystal data

C_13_H_11_BBrFO_3_
*M* *_r_* = 324.94
Monoclinic, 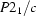

*a* = 14.913 (2) Å
*b* = 4.0214 (6) Å
*c* = 21.945 (3) Å
β = 101.572 (13)°
*V* = 1289.3 (3) Å^3^
*Z* = 4
Mo *K*α radiation
μ = 3.20 mm^−1^
*T* = 100 K
0.18 × 0.16 × 0.08 mm

#### Data collection

Kuma KM-4-CCD diffractometer
Absorption correction: numerical (*CrysAlis RED*; Oxford Diffraction, 2001 ▶) *T*_min_ = 0.588, *T*_max_ = 0.892
18281 measured reflections
2263 independent reflections
1487 reflections with *I* > 2σ(*I*)
*R*_int_ = 0.085

#### Refinement

*R*[*F* ^2^ > 2σ(*F* ^2^)] = 0.039
*wR*(*F* ^2^) = 0.055
*S* = 0.95
2263 reflections
208 parameters
1 restraint
H atoms treated by a mixture of independent and constrained refinement
Δρ_max_ = 0.41 e Å^−3^
Δρ_min_ = −0.42 e Å^−3^

### 

Data collection: *CrysAlis CCD* (Oxford Diffraction, 2001 ▶); cell refinement: *CrysAlis RED* (Oxford Diffraction, 2001 ▶); data reduction: *CrysAlis RED*; program(s) used to solve structure: *SHELXS97* (Sheldrick, 2008 ▶); program(s) used to refine structure: *SHELXL97* (Sheldrick, 2008 ▶); molecular graphics: *DIAMOND* (Brandenburg, 1999 ▶); software used to prepare material for publication: *SHELXL97*.

## Supplementary Material

Crystal structure: contains datablocks I, global. DOI: 10.1107/S1600536809033376/wm2251sup1.cif

Structure factors: contains datablocks I. DOI: 10.1107/S1600536809033376/wm2251Isup2.hkl

Additional supplementary materials:  crystallographic information; 3D view; checkCIF report

## Figures and Tables

**Table 1:** Hydrogen-bond geometry (Å, °)

*D*—H⋯*A*	*D*—H	H⋯*A*	*D*⋯*A*	*D*—H⋯*A*
O1—H1*O*⋯O2^i^	0.84	1.97	2.797 (3)	169
O2—H2*O*⋯O3	0.84	2.03	2.753 (3)	143

Symmetry code: (i) 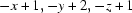.

**Fig. 1.**
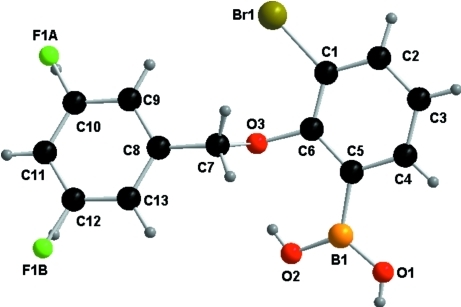
The molecular structure of (I), showing the atom labelling scheme. Displacement ellipsoids for all non-H atoms are drawn at the 50% probability level. H atoms are given as spheres of arbitrary radius.

**Fig. 2.**
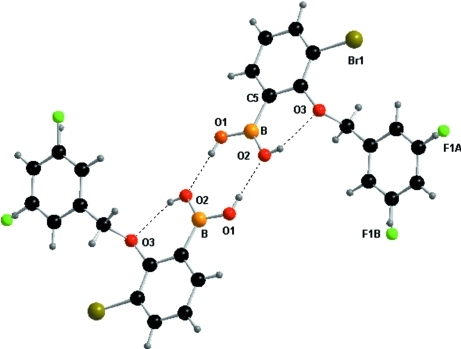
The hydrogen bonding pattern (dashed lines) for the title compound.

## supplementary crystallographic information

### Comment

The high synthetic utility of boronic acids (Bien *et al.*, 1995; Miyaura & Suzuki, 1995) enforces a continuous progress in the preparation and characterization of these compounds. The molecular structure of the title compound, C_13_H_11_BBrFO_3_ (I), is shown in Fig. 1. It is the second example of an arylboronic acid based on the aryl-benzyl ether structure containing an aryloxymethylene substituent. Aryl-benzyl ethers found recently a new application as human immunodeficiency virus-1 (HIV-1) inhibitors (Dai *et al.*, 2009).

The molecular structure of (I) shows that the dioxyboron fragment formed by B, O1 and O2 atoms is essentially planar with the phenyl ring to which the boron atom is connected (C6—C5—B1—O2 = 3.6 (6)°). The hydrogen atom bonded to O2 is involved in an intramolecular O—H···O interaction with atom O3, forming a five-membered ring. The hydrogen atom bonded to O1 is involved in an intermolecular hydrogen bonding to form a centrosymmetric dimer (Fig. 2). The angle between planes formed by two phenyl rings in the same molecule is 14.8 (2)°.

For the structural characterization of a related boronic acid derivative, see: Serwatowski *et al.* (2006).

### Experimental

3-Bromo-2-(3-fluorobenzyloxy)phenylboronic acid was obtained from Aldrich and recrystallized from toluene.

### Refinement

The fluorine atom is disordered over two positions with site occupation factors of 0.325 (6) and 0.675 (6). Positions of most of the hydrogen atoms were refined freely with *U*_iso_(H) = 1.2 or 1.5Ueq(C). The OH hydrogen atoms were refined with a constrained bond length of O—H = 0.84 Å. Hydrogen atoms that belong to the disordered part of the phenyl ring were not refined but added geometrically with a fixed bond length of 0.95 Å.

### Crystal data

**Table:**

C_13_H_11_BBrFO_3_	*F*(000) = 648
*M**_r_* = 324.94	*D*_x_ = 1.674 Mg m^−^^3^
Monoclinic, *P*2_1_/*c*	Mo *K*α radiation, λ = 0.71073 Å
Hall symbol: -P 2ybc	Cell parameters from 10000 reflections
*a* = 14.913 (2) Å	θ = 1.5–29.7°
*b* = 4.0214 (6) Å	µ = 3.20 mm^−^^1^
*c* = 21.945 (3) Å	*T* = 100 K
β = 101.572 (13)°	Plate, colourless
*V* = 1289.3 (3) Å^3^	0.18 × 0.16 × 0.08 mm
*Z* = 4	

### Data collection

**Table:**

Kuma KM-4-CCD diffractometer	2263 independent reflections
Radiation source: fine-focus sealed tube	1487 reflections with *I* > 2σ(*I*)
graphite	*R*_int_ = 0.085
Detector resolution: 8.6479 pixels mm^-1^	θ_max_ = 25.0°, θ_min_ = 3.0°
ω scans	*h* = −17→17
Absorption correction: numerical (*CrysAlis RED*; Oxford Diffraction, 2001)	*k* = −4→4
*T*_min_ = 0.588, *T*_max_ = 0.892	*l* = −26→26
18281 measured reflections	

### Refinement

**Table:**

Refinement on *F*^2^	Primary atom site location: structure-invariant direct methods
Least-squares matrix: full	Secondary atom site location: difference Fourier map
*R*[*F*^2^ > 2σ(*F*^2^)] = 0.039	Hydrogen site location: inferred from neighbouring sites
*wR*(*F*^2^) = 0.055	H atoms treated by a mixture of independent and constrained refinement
*S* = 0.95	*w* = 1/[σ^2^(*F*_o_^2^) + (0.0124*P*)^2^] where *P* = (*F*_o_^2^ + 2*F*_c_^2^)/3
2263 reflections	(Δ/σ)_max_ = 0.001
208 parameters	Δρ_max_ = 0.41 e Å^−^^3^
1 restraint	Δρ_min_ = −0.42 e Å^−^^3^

### Special details

**Table:**

Geometry. All e.s.d.'s (except the e.s.d. in the dihedral angle between two l.s. planes) are estimated using the full covariance matrix. The cell e.s.d.'s are taken into account individually in the estimation of e.s.d.'s in distances, angles and torsion angles; correlations between e.s.d.'s in cell parameters are only used when they are defined by crystal symmetry. An approximate (isotropic) treatment of cell e.s.d.'s is used for estimating e.s.d.'s involving l.s. planes.
Refinement. Refinement of *F*^2^ against ALL reflections. The weighted *R*-factor *wR* and goodness of fit *S* are based on *F*^2^, conventional *R*-factors *R* are based on *F*, with *F* set to zero for negative *F*^2^. The threshold expression of *F*^2^ > σ(*F*^2^) is used only for calculating *R*-factors(gt) *etc*. and is not relevant to the choice of reflections for refinement. *R*-factors based on *F*^2^ are statistically about twice as large as those based on *F*, and *R*- factors based on ALL data will be even larger.

### Fractional atomic coordinates and isotropic or equivalent isotropic displacement parameters (Å^2^)

**Table:**

	*x*	*y*	*z*	*U*_iso_*/*U*_eq_	Occ. (<1)
Br1	0.96580 (3)	1.14409 (10)	0.67991 (2)	0.04384 (17)	
F1A	0.8311 (4)	1.4084 (19)	0.8860 (3)	0.053 (3)	0.325 (6)
F1B	0.5319 (2)	1.0376 (7)	0.82343 (17)	0.0509 (15)	0.675 (6)
O1	0.59426 (15)	0.7687 (6)	0.46913 (11)	0.0312 (7)	
H1O	0.5381	0.8110	0.4639	0.047*	
O2	0.59005 (15)	1.0479 (6)	0.56380 (11)	0.0303 (7)	
H2O	0.6270	1.1287	0.5941	0.045*	
O3	0.75692 (15)	1.0863 (6)	0.64438 (12)	0.0274 (7)	
C1	0.8937 (3)	0.9636 (8)	0.6059 (2)	0.0298 (11)	
C2	0.9361 (3)	0.8402 (10)	0.5612 (2)	0.0371 (12)	
C3	0.8850 (3)	0.7134 (9)	0.5063 (2)	0.0343 (12)	
C4	0.7900 (3)	0.7262 (9)	0.4969 (2)	0.0281 (11)	
C5	0.7446 (2)	0.8543 (9)	0.54136 (17)	0.0216 (9)	
C6	0.7993 (3)	0.9652 (8)	0.59737 (19)	0.0254 (10)	
C7	0.7527 (3)	0.8354 (10)	0.6923 (2)	0.0340 (12)	
C8	0.7268 (3)	0.9918 (8)	0.74709 (19)	0.0277 (11)	
C9	0.7913 (3)	1.1548 (10)	0.79215 (19)	0.0279 (10)	
C10	0.7665 (3)	1.2884 (9)	0.8437 (2)	0.0363 (12)	
H10	0.8111	1.4046	0.8730	0.044*	0.675 (6)
C11	0.6793 (3)	1.2605 (9)	0.8545 (2)	0.0371 (12)	
C12	0.6166 (3)	1.0960 (11)	0.8101 (2)	0.0432 (12)	
H12	0.5557	1.0732	0.8164	0.052*	0.325 (6)
C13	0.6380 (3)	0.9641 (9)	0.7575 (2)	0.0392 (13)	
B1	0.6376 (3)	0.8885 (11)	0.5250 (2)	0.0242 (11)	
H2	1.001 (2)	0.854 (8)	0.5647 (14)	0.029*	
H3	0.915 (2)	0.627 (8)	0.4740 (14)	0.029*	
H4	0.757 (2)	0.661 (8)	0.4593 (15)	0.029*	
H7A	0.711 (2)	0.663 (8)	0.6717 (14)	0.029*	
H7B	0.814 (2)	0.734 (7)	0.7016 (14)	0.029*	
H9	0.853 (2)	1.172 (8)	0.7849 (14)	0.029*	
H11	0.661 (2)	1.346 (8)	0.8908 (15)	0.029*	
H13	0.592 (2)	0.846 (8)	0.7247 (14)	0.029*	

### Atomic displacement parameters (Å^2^)

**Table:**

	*U*^11^	*U*^22^	*U*^33^	*U*^12^	*U*^13^	*U*^23^
Br1	0.0292 (2)	0.0242 (2)	0.0660 (3)	−0.0001 (2)	−0.0193 (2)	0.0000 (3)
F1A	0.042 (5)	0.071 (6)	0.041 (6)	−0.001 (4)	−0.005 (4)	−0.022 (4)
F1B	0.023 (2)	0.065 (3)	0.061 (3)	0.009 (2)	0.0017 (17)	−0.003 (2)
O1	0.0186 (14)	0.0417 (18)	0.0310 (17)	0.0012 (12)	−0.0005 (13)	−0.0128 (13)
O2	0.0221 (15)	0.0386 (18)	0.0260 (18)	0.0020 (12)	−0.0048 (13)	−0.0083 (14)
O3	0.0284 (15)	0.0150 (15)	0.0336 (18)	0.0036 (12)	−0.0063 (13)	0.0002 (14)
C1	0.023 (2)	0.015 (2)	0.045 (3)	0.0029 (17)	−0.012 (2)	0.005 (2)
C2	0.015 (2)	0.024 (2)	0.069 (4)	0.005 (2)	−0.002 (2)	0.002 (3)
C3	0.026 (3)	0.023 (2)	0.058 (4)	0.0020 (19)	0.020 (2)	−0.005 (2)
C4	0.026 (3)	0.022 (2)	0.035 (3)	−0.0007 (18)	0.003 (2)	0.003 (2)
C5	0.024 (2)	0.0085 (18)	0.030 (3)	0.0010 (19)	0.0004 (19)	0.001 (2)
C6	0.027 (2)	0.013 (2)	0.035 (3)	0.0072 (17)	0.003 (2)	0.0028 (19)
C7	0.038 (3)	0.016 (2)	0.041 (3)	0.002 (2)	−0.008 (2)	0.000 (2)
C8	0.035 (3)	0.011 (2)	0.033 (3)	0.0038 (18)	−0.003 (2)	0.0024 (19)
C9	0.024 (2)	0.022 (2)	0.033 (3)	0.002 (2)	−0.002 (2)	0.005 (2)
C10	0.036 (3)	0.026 (3)	0.043 (3)	0.004 (2)	−0.004 (3)	0.001 (2)
C11	0.038 (3)	0.029 (3)	0.043 (3)	0.013 (2)	0.004 (3)	−0.004 (2)
C12	0.027 (3)	0.037 (3)	0.065 (4)	0.007 (2)	0.006 (3)	−0.003 (3)
C13	0.036 (3)	0.023 (2)	0.049 (4)	−0.001 (2)	−0.012 (3)	−0.007 (2)
B1	0.027 (3)	0.014 (2)	0.031 (3)	−0.003 (2)	0.005 (2)	0.004 (2)

### Geometric parameters (Å, °)

**Table:**

Br1—C1	1.902 (4)	C5—B1	1.569 (5)
O1—B1	1.355 (5)	C7—C8	1.476 (5)
O1—H1O	0.8400	C7—H7A	0.98 (3)
O2—B1	1.371 (5)	C7—H7B	0.98 (3)
O2—H2O	0.8400	C8—C13	1.393 (5)
O3—C6	1.401 (4)	C8—C9	1.397 (5)
O3—C7	1.467 (4)	C9—C10	1.368 (5)
C1—C2	1.362 (5)	C9—H9	0.96 (3)
C1—C6	1.384 (5)	C10—C11	1.372 (5)
C2—C3	1.387 (5)	C10—H10	0.9500
C2—H2	0.96 (3)	C11—C12	1.376 (5)
C3—C4	1.392 (5)	C11—H11	0.95 (3)
C3—H3	0.97 (3)	C12—C13	1.365 (5)
C4—C5	1.394 (5)	C12—H12	0.9500
C4—H4	0.91 (3)	C13—H13	1.01 (3)
C5—C6	1.404 (5)		
			
B1—O1—H1O	109.5	O3—C7—H7B	105.2 (19)
B1—O2—H2O	109.5	C8—C7—H7B	112.9 (19)
C6—O3—C7	112.3 (3)	H7A—C7—H7B	106 (3)
C2—C1—C6	120.7 (4)	C13—C8—C9	117.6 (4)
C2—C1—Br1	119.3 (3)	C13—C8—C7	120.8 (4)
C6—C1—Br1	119.9 (3)	C9—C8—C7	121.4 (4)
C1—C2—C3	120.4 (4)	C10—C9—C8	120.4 (4)
C1—C2—H2	122 (2)	C10—C9—H9	122 (2)
C3—C2—H2	117 (2)	C8—C9—H9	117.4 (19)
C2—C3—C4	118.8 (4)	C9—C10—C11	122.3 (4)
C2—C3—H3	121.1 (19)	C9—C10—H10	118.9
C4—C3—H3	120.1 (19)	C11—C10—H10	118.9
C3—C4—C5	122.2 (4)	C10—C11—C12	116.7 (4)
C3—C4—H4	118 (2)	C10—C11—H11	123 (2)
C5—C4—H4	120 (2)	C12—C11—H11	120 (2)
C4—C5—C6	116.9 (3)	C13—C12—C11	123.0 (5)
C4—C5—B1	119.1 (3)	C13—C12—H12	118.5
C6—C5—B1	123.9 (4)	C11—C12—H12	118.5
C1—C6—O3	120.0 (4)	C12—C13—C8	119.9 (4)
C1—C6—C5	120.9 (4)	C12—C13—H13	123.2 (19)
O3—C6—C5	119.1 (3)	C8—C13—H13	116.9 (19)
O3—C7—C8	110.3 (3)	O1—B1—O2	121.1 (3)
O3—C7—H7A	105.8 (19)	O1—B1—C5	117.0 (4)
C8—C7—H7A	115.6 (19)	O2—B1—C5	121.8 (4)
			
C6—C1—C2—C3	−0.1 (6)	C6—O3—C7—C8	167.5 (3)
Br1—C1—C2—C3	−178.7 (3)	O3—C7—C8—C13	102.5 (4)
C1—C2—C3—C4	2.4 (6)	O3—C7—C8—C9	−81.5 (4)
C2—C3—C4—C5	−1.7 (6)	C13—C8—C9—C10	−1.6 (5)
C3—C4—C5—C6	−1.3 (5)	C7—C8—C9—C10	−177.8 (4)
C3—C4—C5—B1	174.8 (3)	C8—C9—C10—C11	2.1 (6)
C2—C1—C6—O3	178.6 (3)	C9—C10—C11—C12	−1.3 (6)
Br1—C1—C6—O3	−2.8 (4)	C10—C11—C12—C13	0.1 (6)
C2—C1—C6—C5	−3.0 (5)	C11—C12—C13—C8	0.3 (6)
Br1—C1—C6—C5	175.6 (3)	C9—C8—C13—C12	0.4 (5)
C7—O3—C6—C1	−82.8 (4)	C7—C8—C13—C12	176.6 (4)
C7—O3—C6—C5	98.8 (3)	C4—C5—B1—O1	4.4 (5)
C4—C5—C6—C1	3.6 (5)	C6—C5—B1—O1	−179.9 (3)
B1—C5—C6—C1	−172.3 (3)	C4—C5—B1—O2	−172.2 (3)
C4—C5—C6—O3	−178.0 (3)	C6—C5—B1—O2	3.6 (6)
B1—C5—C6—O3	6.1 (5)		

### Hydrogen-bond geometry (Å, °)

**Table:**

*D*—H···*A*	*D*—H	H···*A*	*D*···*A*	*D*—H···*A*
O1—H1O···O2^i^	0.84	1.97	2.797 (3)	169
O2—H2O···O3	0.84	2.03	2.753 (3)	143

Symmetry codes: (i) −*x*+1, −*y*+2, −*z*+1.

## References

[bb1] Bien, J. T., Shang, M. & Smith, B. D. (1995). *J. Org. Chem* **60**, 2147–2152.

[bb2] Brandenburg, K. (1999). *DIAMOND* Crystal Impact GbR, Bonn, Germany.

[bb3] Dai, H. L., Liu, W. Q., Xu, H., Yang, L. M., Lv, M. & Zheng, Y. T. (2009). *Chem. Pharm. Bull.* **57**, 84–86. 10.1248/cpb.57.84 19122322

[bb4] Serwatowski, J., Klis, T. & Kacprzak, K. (2006). *Acta Cryst.* E**62**, o1308-o1309.

[bb5] Miyaura, N. & Suzuki, A. (1995). *Chem. Rev* **95**, 2457–2483.

[bb6] Oxford Diffraction (2001). *CrysAlis CCD* and *CrysAlis RED* Oxford Diffraction Poland, Wrocław, Poland.

[bb7] Sheldrick, G. M. (2008). *Acta Cryst.* A**64**, 112–122. 10.1107/S0108767307043930 18156677

